# The Metabolic and Hepatic Impact of Two Personalized Dietary Strategies in Subjects with Obesity and Nonalcoholic Fatty Liver Disease: The Fatty Liver in Obesity (FLiO) Randomized Controlled Trial

**DOI:** 10.3390/nu11102543

**Published:** 2019-10-22

**Authors:** Bertha Araceli Marin-Alejandre, Itziar Abete, Irene Cantero, J. Ignacio Monreal, Mariana Elorz, José Ignacio Herrero, Alberto Benito-Boillos, Jorge Quiroga, Ana Martinez-Echeverria, Juan Isidro Uriz-Otano, María Pilar Huarte-Muniesa, Josep A. Tur, J. Alfredo Martinez, M. Angeles Zulet

**Affiliations:** 1 Department of Nutrition, Food Sciences and Physiology and Centre for Nutrition Research, Faculty of Pharmacy and Nutrition, University of Navarra, 31008 Pamplona, Spain; bmarin.1@alumni.unav.es (B.A.M.-A.); icgonzalez@unav.es (I.C.); 2 Biomedical Research Centre Network in Physiopathology of Obesity and Nutrition (CIBERobn), Instituto de Salud Carlos III, 28029 Madrid, Spain; pep.tur@uib.es; 3 Navarra Institute for Health Research (IdiSNA), 31008 Pamplona, Spain; jimonreal@unav.es (J.I.M.); marelorz@unav.es (M.E.); iherrero@unav.es (J.I.H.); albenitob@unav.es (A.B.-B.); jquiroga@unav.es (J.Q.); ana.martinez.echeverria@cfnavarra.es (A.M.-E.); jurizota@cfnavarra.es (J.I.U.-O.); phuartem@cfnavarra.es (M.P.H.-M.); 4 Clinical Chemistry Department, Clinica Universidad de Navarra 31008, Pamplona, Spain; 5 Department of Radiology, Clinica Universidad de Navarra, 31008 Pamplona, Spain; 6 Liver Unit, Clinica Universidad de Navarra 31008, Pamplona, Spain; 7 Centro de Investigación Biomédica en Red de Enfermedades Hepáticas y Digestivas (CIBERehd), 28029 Madrid, Spain; 8 Department of Internal Medicine, Clinica Universidad de Navarra, 31008 Pamplona, Spain; 9 Department of Gastroenterology, Complejo Hospitalario de Navarra, 31008 Pamplona, Spain; 10 Research Group on Community Nutrition and Oxidative Stress, University of Balearic Islands, 07122 Palma, Spain

**Keywords:** Obesity, NAFLD, dietary intervention, AHA, FLiO, fatty liver, Mediterranean Diet

## Abstract

The prevalence of nonalcoholic fatty liver disease (NAFLD) is increasing worldwide. NAFLD management is mainly focused on weight loss, but the optimal characteristics of the diet demand further investigation. This study aims to evaluate the effects of two personalized energy-restricted diets on the liver status in overweight or obese subjects with NAFLD after a 6 months follow-up. Ninety-eight individuals from the Fatty Liver in Obesity (FLiO) study were randomized into two groups and followed different energy-restricted diets. Subjects were evaluated at baseline and after 6 months. Diet, anthropometry, body composition, and biochemical parameters were evaluated. Liver assessment included ultrasonography, Magnetic Resonance Imaging, elastography, and determination of transaminases. Both dietary groups significantly improved their metabolic and hepatic markers after the intervention, with no significant differences between them. Multivariate regression models evidenced a relationship between weight loss, adherence to the Mediterranean Diet (MedDiet), and a decrease in liver fat content, predicting up to 40.9% of its variability after 6 months. Moreover, the antioxidant capacity of the diet was inversely associated with liver fat content. Participants in the group with a higher adherence to the MedDiet showed a greater reduction in body weight, total fat mass, and hepatic fat. These results support the benefit of energy-restricted diets, high adherence to the MedDiet, and high antioxidant capacity of the diet for the management of NAFLD in individuals with overweight or obesity.

## 1. Introduction

Nonalcoholic fatty liver disease (NAFLD) is defined as an excessive accumulation of hepatic fat in the absence of consumption of significant amounts of alcohol or other related causes of secondary hepatic steatosis [1,2]. The worldwide prevalence of NAFLD is rising parallelly to the obesity burden and type 2 diabetes mellitus epidemics [3,4]. The pathogenesis of NAFLD is complex, and multiple environmental and genetic factors are involved in its development and progression [5]. The background of genetic predisposition may include variants in genes, such as the Patatin-like phospholipase domain-containing 3 (PNPLA3) or the Transmembrane 6 superfamily member 2 (TM6SF2) [5,6]. Other important factors involved are obesity, dietary intake, physical inactivity, insulin resistance, gut microbiota dysbiosis, epigenetic impairments, among others [4,7,8]. Indeed, NAFLD is considered not only a liver disturbance but also a multisystem disease that is related to type 2 diabetes mellitus, cardiovascular disease, and chronic kidney disease [9]. This condition can lead to nonalcoholic steatohepatitis (NASH), fibrosis, cirrhosis, and, finally, hepatocellular carcinoma [10], which will be the leading cause for liver transplantation in the next few years [11].

Regarding the management of NAFLD, lifestyle modifications focused on weight loss remain the cornerstone of therapy [12]. Strategies like changes in dietary patterns and exercise have demonstrated benefits for the prevention of the onset and progression of this morbid condition [13,14]. Caloric restriction apparently plays a major role in weight loss and in hepatic fat reduction, so it is considered a central element in nutritional interventions for subjects with NAFLD [15]. Currently, 3%–5% loss in body weight is often recommended to improve liver steatosis, with greater medical improvements of liver status when the weight loss is higher [2,16]. However, the evidence for nutritional factors, characteristics of the diet, and dietary strategies for NAFLD treatment remains inconclusive and demands further investigation [14,15,17].

In this context, the objective of this study was to evaluate the effects of two personalized energy-restricted dietary strategies differing in several dietary factors (macronutrients, fiber, meal frequency, total antioxidant capacity, and Mediterranean diet adherence) on liver status, as assessed by imaging techniques and biochemical markers in overweight and obese subjects with NAFLD after a 6 months follow-up.

## 2. Materials and Methods

### 2.1. Study Participants

**Figure 1:**
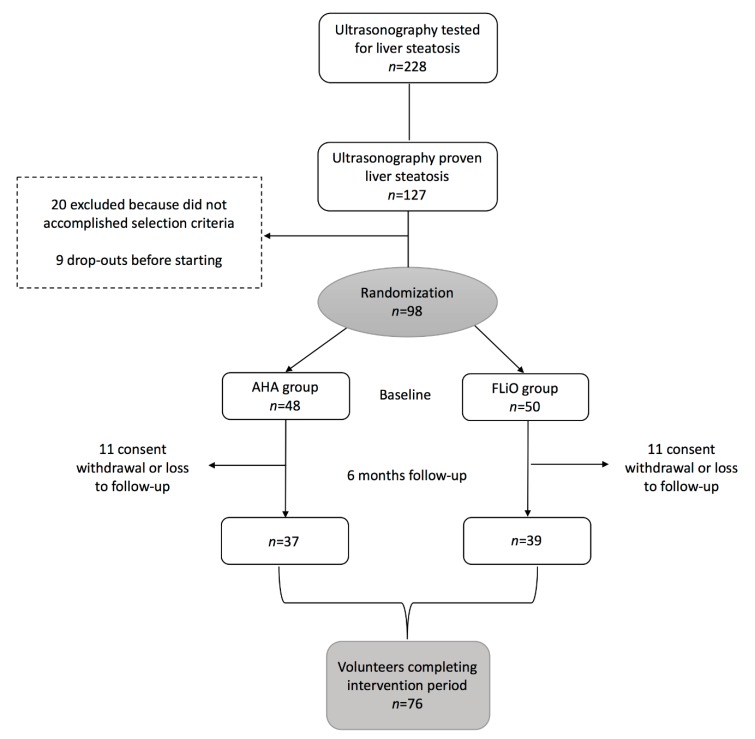
Flowchart of participants in the Fatty Liver in Obesity (FLiO) study.

Ninety-eight (55 Male and 43 Female) overweight or obese (BMI ≥ 27.5 kg/m^2^ to < 40 kg/m^2^) adults between 40–80 years old and with hepatic steatosis confirmed by abdominal ultrasonography were enrolled in the study. A total of 76 of the participants completed the evaluation after 6 months (Figure 1). Exclusion criteria included the presence of known liver disease other than NAFLD, ≥3 kg of body weight loss in the last 3 months, excessive alcohol consumption (>21 and >14 units of alcohol per week for men and women, respectively [18], endocrine disorders (hyperthyroidism or uncontrolled hypothyroidism), pharmacological treatment with immunosuppressants, cytotoxic agents, systemic corticosteroids (or other drugs that could potentially cause hepatic steatosis or altering liver tests) [2], active autoimmune diseases or requiring pharmacological treatment, the use of weight modifiers and severe psychiatric disorders, and the lack of autonomy or an inability to follow the diet, as well as difficulties in following the scheduled visits. This information was obtained in a clinical interview of the subjects before their participation in the study. Each individual gave written informed consent prior to their enrollment. All the procedures performed were in accordance with the Declaration of Helsinki. This trial was approved by the Research Ethics Committee of the University of Navarra (ref. 54/2015) and appropriately registered in www.clinicaltrails.gov (FLiO: Fatty Liver in Obesity study; NCT03183193).

### 2.2. Study Protocol

This randomized controlled trial was designed to compare the effects of two dietary strategies for weight loss with different nutritional characteristics for hepatic status, as well as for anthropometric measurements, body composition, and biochemical markers, in overweight or obese subjects with ultrasonography proven liver steatosis. The intervention had a duration of 6 months, and the participants were randomly assigned to the American Heart Association (AHA) or the Fatty Liver in Obesity (FLiO) group (Figure 1). A comprehensive assessment was carried out at the baseline and at the endpoint of the study, including anthropometric measurements, body composition by dual energy X-Ray absorptiometry (DXA), biochemical determinations, and evaluation of the liver using ultrasonography, Acoustic Radiation Force Impulse (ARFI) elastography, and Magnetic Resonance Imaging (MRI). The imaging evaluations of the liver were performed by a qualified radiologist and hepatologist at the University of Navarra Clinic. Additionally, fasting blood samples were properly collected, processed, and stored at −80 °C for further analyses. A step-based physical activity recommendation of 10,000 steps/day was given to the participants [19]. Physical activity was estimated using the validated Spanish version of the Minnesota Leisure-Time Physical Activity Questionnaire [20]. The energy expenditure in physical activity was estimated assuming the value of 1 MET = 3.5 mL/kg/min, as previously described [21], and the ratio between energy intake and energy expenditure was computed.

### 2.3. Dietary Intervention

Two energy-restricted diets were prescribed and compared. Both diets applied a 30% energy restriction of the total energy requirements of each participant in order to achieve a loss of at least 3%–5% of the initial body weight, according to the objective of the American Association for the Study of Liver Diseases (AASLD) [2]. Resting metabolic rate was calculated using the equation of the Institute of Medicine, as elsewhere described [22], in order to estimate the energy prescription for each subject. The control diet was based on the guidelines of the American Heart Association (AHA) [23], which suggest 3–5 meals/day, with a conventionally balanced distribution of macronutrients (50%–55% of total caloric value from carbohydrates, 15% from proteins, and 30% from lipids) and a healthy fatty acid profile. On the other hand, the FLiO diet was designed with a higher meal frequency (7 meals/day), including breakfast, lunch, dinner, two snacks in the morning, and two snacks in the afternoon. The established macronutrient distribution was 40%–45% of the total caloric value from carbohydrates (low glycemic index), 25% from proteins (mainly from vegetable sources), and 30%–35% from lipids (extra virgin olive oil and Omega-3 fatty acids to the detriment of saturated and trans fats). Moreover, the dietary pattern included a high adherence to the Mediterranean Diet (MedDiet), subsequently involving an increased quantity of natural antioxidants [24]. Participants were provided with a 7 days menu plan in both groups. A semiquantitative food frequency questionnaire (FFQ) of 137 items previously validated in Spain for energy and nutrient intake [25] was used to assess the diets of the participants at baseline and after 6 months of the study. The mean antioxidant capacity values of the foods contained in each item of the FFQ were used to estimate the total antioxidant capacity (TAC) of the diet, as previously described [26]. The adherence to the MedDiet was assessed with a 17-point screening questionnaire, with a final score ranging from 0 to 17 and a higher score indicating a better adherence to the MedDiet [27].

### 2.4. Anthropometric, Body Composition and Biochemical Assessment

The determination of anthropometric measurements (body weight, height, and waist circumference), body composition by DXA (Lunar iDXA, encore 14.5, Madison, WI, USA), and blood pressure (Intelli Sense. M6, OMRON Healthcare, Hoofddorp, the Netherlands) was carried out under fasting conditions at the Metabolic Unit of the University of Navarra following standardized procedures, as previously described [28]. Body Mass Index (BMI) was calculated as the body weight divided by the squared height (kg/m^2^). Biochemical determinations, including blood glucose, aspartate aminotransferase (AST), alanine aminotransferase (ALT), gamma glutamyl transferase (GGT), total cholesterol (TC), high-density lipoprotein cholesterol (HDL-c), low density lipoprotein cholesterol (LDL-c), and triglyceride (TG) concentrations were measured on an autoanalyzer Pentra C-200 (HORIBA ABX, Madrid, Spain) with specific commercial kits. Insulin, C-reactive protein (CRP), leptin, and adiponectin concentrations were measured using specific ELISA kits (Demeditec; Kiel-Wellsee, Germany) in a Triturus autoanalyzer (Grifols, Barcelona, Spain). Insulin resistance was estimated using the Homeostasis Model Assessment Index (HOMA-IR), which was calculated using the formula elsewhere described [28]. Finally, the Fatty Liver Index (FLI) was computed using serum TG, BMI, waist circumference, and GGT concentrations, as previously published [29]. FLI values <30 rule out liver steatosis and values ≥60 indicate liver steatosis [30].

### 2.5. Imaging Techniques for the Assessment of Liver Status

The entire hepatic assessment was performed under fasting conditions by qualified staff at the University of Navarra Clinic. Ultrasonography (Siemens ACUSON S2000 and S3000) was carried out to determine the presence of hepatic steatosis in accordance with the previously described methodology [31,32,33]. In addition, ARFI elastography was performed along with the ultrasonography in order to assess liver stiffness. The median value of liver stiffness was obtained from the ten valid ARFI measurements that were performed on each participant [34]. The same experienced radiologist executed all the ultrasonographic evaluations at the department of Ultrasonography and Radiology. Finally, an MRI (Siemens Aera 1.5 T) was used to determine the hepatic volume and the fat content of the liver (Dixon technique), as described elsewhere [31].

### 2.6. Statistical Analyses

The sample size was calculated with weight loss as a primary outcome, given that the current recommendations of the AASLD are focused on weight loss to ameliorate NAFLD features [2]. In this sense, and according to previous studies [28], the sample size was calculated to detect a difference of 1.0 (1.5 kg) between the AHA and FLiO groups in their reduction of weight, with a 95% confidence interval (α = 0.05) and a statistical power of 80% (*β* = 0.8). This approach estimated a total of 36 participants per study group but considering the estimated dropout rate of 20%–30% (according to the experience of the research group), 50 subjects were included in each group. However, two subjects were excluded from the AHA group due to important alterations in the initial evaluation of biochemical parameters, which indicated that they required medical treatment. Consequently, this trail started with 98 subjects (*n* = 48 in AHA group and *n* = 50 in FLiO group). The mean value (standard deviation) is reported for the studied variables. The normality of the distribution of the evaluated variables was assessed by the Shapiro–Wilk test. The differences between the groups were compared by means of Student’s *t*-test or the Mann–Whitney *U* test when appropriate. The differences between the beginning and the end of the intervention period within each group were analyzed by a paired Student’s *t*-test or Wilcoxon signed-rank test when appropriate. Categorical variables were compared using a Chi-squared test. Linear regression analyses were used to evaluate the potential association between the anthropometry, body composition, and components of the diet with the hepatic status variables. Multivariate linear regression models were adjusted for potential confounders considering the group of intervention, age, sex, physical activity, and energy intake. The median value of the MedDiet Adherence questionnaire was used to classify the participants into low adherence (<50th percentile) or high adherence (≥50th percentile). The differences between the two groups were assessed by Student’s *t*-test or a Mann–Whitney *U* test. Analyses were carried out using Stata version 12.0 software (StataCorp, College Station, TX, USA). All *p*-values presented are two-tailed and were considered statistically significant at *p* < 0.05.

## 3. Results

After 6 months of nutritional intervention, a total of 76 participants completed the evaluation. Both the AHA and FLiO groups exhibited a significant body weight loss (−9.7% (5.0) vs. −10.1% (6.5); *p* = 0.757). Furthermore, BMI and the rest of the anthropometrical measurements were significantly lowered in both dietary groups. No statistically significant differences were found between the intervention groups for these variables (Table 1). Regarding the biochemical parameters, both dietary groups showed improvements in total cholesterol, high-density lipoprotein cholesterol (HDL-c), low-density lipoprotein cholesterol (LDL-c), and triglycerides concentrations. Fasting glucose, insulin, HOMA-IR, and leptin had a significant reduction in both groups, while adiponectin and C-reactive protein only showed significant improvements in the FLiO group (Table 1). However, the changes from baseline to 6 months of intervention for all these variables did not differ between both dietary strategies.

MRI evidenced a significant reduction in hepatic volume and hepatic fat content after 6 months of follow-up in both groups (Table 2). A significant reduction in liver enzymes (AST, ALT, and GGT) was observed in both groups, with the exception of AST, which significantly decreased only in the AHA group (Table 2). On the other hand, liver stiffness did not show a significant change after this period in any of the groups (Table 2). A significant reduction was found in the Fatty Liver Index with both dietary interventions. Notably, there were no mentionable statistical differences in the changes of liver markers between the groups (Table 2).

Furthermore, there were no significant differences at baseline in relation to dietary intake for both dietary groups (Table 3). As expected, the changes in meal frequency, percentage of protein, PUFA, and TAC were significantly higher in the FLiO group, while the percentage of carbohydrates significantly decreased. Notably, the adherence to the MedDiet significantly increased in both groups, but this change was significantly higher (*p* = 0.002) in the FLiO group. Similar results were found when the intake at 6 months was compared. Regarding energy intake, there was a significant difference between both groups at 6 months. However, the change from baseline to the endpoint was not statistically significant between them. On the other hand, physical activity significantly increased in both intervention groups, but the difference between them (*p* = 0.326) was not statistically significant (Table 3). When comparing the ratio of energy intake to the energy expenditure in physical activity, there was no significant difference between the groups (Table 3).

Given that the changes in anthropometric clinical and biochemical parameters did not statistically differ between AHA and FLiO groups and that both approaches displayed similar outcomes for relevant variables of liver status, we considered both dietary strategies as equally nutritionally effective (Table 2 and Table 3), and, consequently, the two groups were merged and evaluated as one sample for the subsequent analyses.

Linear regression analyses (adjusted by group of intervention, age, sex, 6 months physical activity, and 6 months energy intake) were performed in order to evaluate the factors potentially involved with hepatic status parameters after the 6 months of the dietary intervention (Table 4). Notably, weight loss percentage was significantly associated with improvements in ALT, a reduction of liver fat percentage, FLI, and liver stiffness. Parallelly, the decrease in total fat mass was significantly associated with a reduction in liver fat content, FLI, and liver stiffness, while the decrease in visceral adipose tissue seemed to influence liver stiffness. Regarding dietary factors, the increase in the adherence to MedDiet seemed to play a role in the decrease of liver fat percentage, while its association with lower values of ALT and liver stiffness was only marginally significant. Additionally, the decrease in the lipid percentage of the diet was associated with a reduction in hepatic fat content. Finally, the change in dietary fiber consumption was inversely associated with FLI.

To explore this issue in a more comprehensive manner, a linear regression analysis was used. The effect of nutritional factors on liver fat content after 6 months of dietary treatment was analyzed by linear univariate regression and subsequently a multivariate analysis (Table S1). In addition to the global MedDiet adherence, the TAC of the diet was the only nutritional factor that showed a consistent, significant, and inverse association with liver fat content.

According to the relationships observed in the previous analyses, the influence of weight loss, adherence to MedDiet, and TAC on liver fat content is summarized in Table 5. Models of these main factors that are unadjusted and adjusted by potential confounders are presented. In model 5 and model 6, both weight loss and adherence to the MedDiet were included along with the rest of the adjusting variables. In model 5, MedDiet adherence remained statistically significant, while in model 6, both variables showed statistical significance and explained up to 40.9% of the variation in liver fat content (Adjusted *R*^2^ = 0.409; *p*-model < 0.001) after the 6 months intervention. In model 7 and model 8, the TAC of the diet was included instead of its MedDiet adherence. Model 7 illustrates the inverse association between the TAC and liver fat content, even after an adjustment for weight loss, and explains up to 30.5% of the variation in liver fat (Adjusted *R*^2^ = 0.305; *p*-model < 0.001).

**Figure 2:**
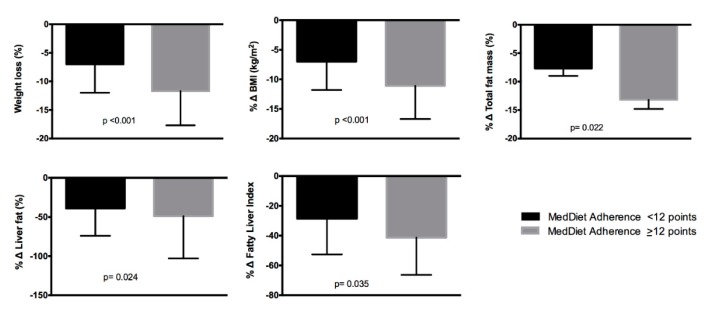
Weight loss percentage and percentage of changes in selected variables according to the median score of the Mediterranean Diet (MedDiet) adherence questionnaire (<12 points *n* = 30 and > 12 points *n* = 46) after 6 months of dietary intervention.

Because the adherence to the MedDiet was an important dietary factor significantly associated with an improvement in hepatic status variables even after multiple adjustments in the regression analyses, the whole sample was categorized into two groups according to the median value of the adherence to the MedDiet questionnaire (low adherence <12 points, *n* = 30, and high adherence ≥12 points, *n* = 46). The percentage of changes in anthropometric and hepatic status variables were compared between these two groups (Figure 2). The group with a higher adherence to the MedDiet showed significantly greater reductions in body weight, BMI, total fat mass, liver fat, and FLI.

## 4. Discussion

This randomized controlled trial compared the effects of two personalized energy-restricted dietary strategies on anthropometry, body composition, biochemical determinations, and the non-invasive parameters of liver status in overweight or obese subjects with ultrasonography proven liver steatosis after 6 months of intervention. The main results evidenced the important association between weight loss, TAC, and MedDiet adherence, with an improvement in hepatic status variables such as liver fat content, FLI, and liver stiffness, regardless of the group of intervention and under the prescription of an energy-restricted balanced diet.

The pathogenesis of NAFLD involves a variety of components where dietary factors seem to be of key importance and have been associated with weight gain, obesity, and NAFLD development. Such factors include a high intake of calories and an excessive consumption of saturated fats, refined carbohydrates, and fructose, all distinctive features of the Western diet [35,36]. The important association between NAFLD and obesity emphasizes the role of dietary intake in the development and progression of NAFLD, since its prevalence rises with an increase in BMI [4]; in general, weight gain leads to the accumulation of fat in the liver [37]. Accordingly, lifestyle interventions focusing on weight loss by means of a reduction in caloric intake and/or an increase in energy expenditure are currently the first line treatment for subjects with NAFLD [2,38,39].

In the present study, both groups achieved comparable results in the evaluated variables, including weight loss, and the reduction in liver fat content and hepatic volume was evaluated by MRI, transaminases, and FLI. The findings with no significant differences in the changes between the intervention groups (even when dietary intake presented the expected statistical differences) may be explained by the energy restriction of 30% applied to both diets and the consequently similar weight loss of more than 9% in both groups, which agrees with the objectives proposed by the AASLD for the treatment of subjects with NAFLD. The AASLD recommends a weight loss of at least 3%–5% of body weight to reduce liver steatosis, while a loss of 7%–10% may be needed to improve fibrosis and the other histological characteristics of NASH [2,40]. Moreover, both dietary strategies in this trail were carefully designed and based on previously studied healthy dietary approaches, which have shown beneficial effects in the management of cardiovascular risk factors. The AHA diet was based on the AHA guidelines, and the FLiO diet was based on the RESMENA diet [28,41], instead of a comparison with a control group without active intervention or receiving only general recommendations.

On the other hand, several authors have proposed that dietary pattern analysis is the most realistic approach to evaluate the associations between diet and disease [42,43]. Accordingly, we evaluated the adherence to the MedDiet, as well as individual dietary features.

The MedDiet has shown a variety of health effects mainly related to the prevention of cardiovascular disease [44]. Thus, its potential repercussions in NAFLD development and treatment continue to be studied. A recent cross-sectional study with more than 13,000 individuals evaluated two population-based cohorts of adults from Switzerland and England and found that a greater adherence to the MedDiet was associated with a lower prevalence of hepatic steatosis [45]. Meanwhile, a study that compared subjects with NAFLD to an age, sex, and BMI matched control group found that a higher adherence to the MedDiet was not associated with a lower likelihood of having NAFLD but was associated with lower insulin resistance and less severe liver disease [46]. Recently, a randomized controlled trail compared a group that only followed a MedDiet, a group that followed a “Mediterranean lifestyle” (diet, exercise, and sleep recommendations), and a group that only received general dietary guidelines. The MedDiet group had significantly lowered liver stiffness values, while the subjects with the “Mediterranean lifestyle” prescription had significantly reduced ALT levels and liver stiffness in comparison with the third group [17]. In our study, most of the individual characteristics of the diet showed a small association with liver status after the dietary intervention, while a greater increase in the TAC and a higher adherence to the MedDiet (along with weight loss) seemed to be more decisive factors for additional benefits in the treatment of subjects with NAFLD. In the regression analyses, the TAC of the diet and the adherence to the MedDiet were significant and inversely associated with less hepatic fat content, even when the models included the percentage of weight loss.

Given that weight reduction through lifestyle changes is not a feasible alternative for all individuals with NAFLD, an increasing number of studies have been carried out to determine if certain dietary or lifestyle modifications might have beneficial effects on liver status even in the absence of weight loss [39,47]. These dietary modifications are focused on following a “high quality” and a “healthy” diet and aim to avoid foods typically present in the Western dietary pattern [15]. In a recent study with a duration of 12 weeks, hepatic steatosis was significantly diminished to a similar degree by both *ad libitum* low-fat and Mediterranean diets, even with only a 2% body weight loss [48].

Regarding liver stiffness, the results of the ARFI elastography did not significantly change in the whole sample after the intervention period and the differences between the groups did not reach statistical significance. Interestingly, liver stiffness decreased only in the FLiO group, although the difference in the changes was only marginally significant (*p* = 0.062). The remission or the stabilization of fibrosis is considered to be a positive aspect in clinical trials with NAFLD subjects [34]. Even when our results were derived from a medium-term intervention (6 months), the period of follow-up may have not been long enough to evidence an improvement in liver stiffness assessed by ARFI elastography. Previous studies have found an improvement in liver stiffness [49] while others have found no changes after dietary treatment [48]. Notably, in the regression analyses, a lower liver stiffness at the endpoint of the intervention was associated with a loss of body weight, visceral adipose tissue, and total fat mass and was marginally associated with a higher adherence to the MedDiet.

Inflammation plays a major role in the evolution of NAFLD; liver steatosis accompanied by hepatic inflammation (NASH) increases the risk of progression to fibrosis and cirrhosis [11]. Moreover, an association between adiponectin levels and liver fibrosis in subjects with chronic liver disease, including those with NAFLD, has been suggested. However, this relationship varies widely among the different liver diseases, and conflicting results are reported in the literature, so the nature of these associations remains poorly understood [50]. On the other hand, there is evidence that weight loss is a central factor in reducing the pro-inflammatory markers in obese or overweight individuals and that a hypocaloric diet has anti-inflammatory effects independent of the diet’s composition [51]. Interestingly, even when the weight loss percentage and the changes in the rest of the variables evaluated in our study were not significantly different between the groups, inflammatory markers (such as adiponectin and C-reactive protein) significantly improved in the FLiO group but not in the AHA group. These results might be related to the higher adherence to the MedDiet and the significant increase of the TAC in subjects following the FLiO diet, which entails a higher intake of fruits and vegetables and a “healthier profile” of fatty acids. A recent review and meta-analysis concluded that a higher intake of fruits and vegetables leads to a reduction in proinflammatory mediators [52]. Thus, the effect of weight loss in inflammatory markers might be greater when accompanied by a higher intake of fruits and vegetables, and we hypothesize that in a longer follow-up, the FLiO diet (with a higher adherence to de MedDiet and a higher TAC) may provide greater improvements. Due to the limited evidence on this issue, more well-designed trails specifically aiming to evaluate the interplay between inflammatory markers and dietary features in the treatment of NAFLD are needed, especially regarding NASH and liver fibrosis.

There are some limitations in this study that should be acknowledged. First, NAFLD was evaluated using non-invasive techniques instead of a liver biopsy, which is currently the most reliable approach for detecting steatohepatitis and fibrosis in NAFLD subjects. Nevertheless, liver biopsy is limited by cost, sampling errors, and procedure-related complications [2]. However, we carried out a compressive evaluation of liver status, and the design of this study was based on validated and widely used imaging techniques for the assessment of liver steatosis (MRI and ultrasonography [33]) and liver stiffness (ARFI elastography [53]). Second, the evaluation of the diet was carried out using self-reported information of the participants by means of an FFQ, which may produce a bias in dietary evaluation. On the other hand, the major strengths of this study include that it is a randomized controlled trial (considered to be the gold standard for evaluating the efficacy and safety of dietary interventions). Additionally, the implemented strategies consisted of personalized diets with individual follow-ups and tended to promote long term behavioral changes and a healthy lifestyle.

## 5. Conclusions

The data in this study suggest that, in the context of energy restriction, both the AHA and the FLiO diets may be valid options for the dietary treatment of NAFLD in overweight or obese subjects. Moreover, a higher TAC and adherence to the MedDiet might provide additional benefits to weight loss in the treatment of obesity and associated comorbidities, such as NAFLD.

## Figures and Tables

**Table 1:** Characteristics at baseline and after 6 months of dietary intervention in subjects with NAFLD according to the group of intervention.

	AHA			FLiO				
	Baseline (*n* = 48)	6 months (*n* = 37)	*p*-Value ^a^	Baseline (*n* = 50)	6 months (*n* = 39)	*p*-Value ^a^	Baseline *p*-Value ^b^	∆ *p*-Value ^c^
Age (years)	51.1 (9.8)		-	49.2 (8.9)		-	0.326	-
Sex (Women/Men)	20/28		-	27/23		-	0.666 ^e^	-
**Anthropometry and body composition**								
Weight (kg)	94.4 (14.4)	84.2 (13.1)	<0.001	95.1 (14.0)	86.6 (13.2)	<0.001	0.791	0.621
BMI (kg/m^2^)	33.7 (4.0)	30.2 (4.1)	<0.001	33.3 (3.5)	30.1 (3.6)	<0.001	0.645	0.929
Waist circumference (cm)	109.7 (9.6)	98.7 (14.8)	<0.001	108.3 (9.2)	99.4 (9.5)	<0.001	0.462	0.614
Total fat mass (%)	42.7 (6.0)	37.0 (7.4)	<0.001	42.3 (6.2)	37.8 (7.8)	<0.001	0.759	0.857
Visceral adipose tissue (kg)	2.5 (1.1)	1.6 (0.8)	<0.001	2.3 (1.1)	1.5 (0.8)	<0.001	0.235	0.330
SBP (mmHg)	133 (14.3)	119 (23.2)	0.003	128 (15.2)	123 (15.3)	0.009	0.089	0.111
DBP (mmHg)	87 (8.2)	79 (7.9)	<0.001	86 (9.3)	82 (8.3)	<0.001	0.797	0.661
**Biochemical parameters**								
Total cholesterol (mg/dL)	192 (40.4)	177 (42.9)	0.089	197 (34.7)	185 (41.3)	0.051	0.514	0.362
HDL cholesterol (mg/dL)	51.9 (14.3)	53.0 (13.8)	0.073	53.8 (12.1)	54.7 (12.0)	0.251	0.436	0.858
LDL cholesterol (mg/dL)	114 (37.3)	103 (36.7)	0.264	118 (30.4)	112 (35.4)	0.265	0.576	0.934
Triglycerides (mg/dL)	128.6 (65.7)	98.6 (41.4)	0.003	129.1 (61.9)	90.6 (58.5)	<0.001	0.960	0.103
Fasting glucose (mg/dL)	103.8 (17.9)	94.7 (14.4)	<0.001	101.4 (13.5)	93.0 (10.8)	<0.001	0.912	0.204
Insulin (mU/L)	17.5 (9.4)	11.2 (7.2)	<0.001	16.6 (7.4)	11.2 (7.3)	<0.001	0.615	0.788
HOMA-IR	4.6 (2.8)	2.7 (2.2)	<0.001	4.2 (2.1)	2.6 (1.9)	<0.001	0.623	0.581
Leptin (ng/mL)	37.1 (27.0)	20.8 (15.7)	<0.001	38.8 (31.1)	22.3 (17.1)	<0.001	0.934	0.770
Adiponectin (µg/mL)	6.7 (2.2)	8.0 (3.0)	0.118	6.6 (2.2)	9.5 (3.7)	<0.001	0.887	0.100
C-reactive protein (mg/dL)	0.65 (1.9)	0.32 (0.4)	0.250	0.40 (0.6)	0.18 (0.2)	<0.001	0.710	0.559

Data are presented as the mean (SD). Nonalcoholic fatty liver disease (NAFLD). American Health Association (AHA). Fatty Liver in Obesity (FLiO). Body Mass Index (BMI). Systolic blood pressure (SBP). Diastolic blood pressure (DBP). High density lipoprotein (HDL). Low density lipoprotein (LDL). Homeostatic Model Assessment for Insulin Resistance (HOMA-IR). ^a^ Comparison within dietary groups (baseline and after 6 months). ^b^ Baseline differences between the AHA and FLiO groups. ^c^ Comparison of changes (baseline and 6 months) between the AHA and FLiO groups. ^e^ Chi-squared test for baseline differences between the AHA and FLiO groups.

**Table 2:** Liver parameters at baseline and after 6 months of dietary intervention in subjects with NAFLD, according to the group of intervention.

	AHA			FLiO				
	Baseline (*n* = 48)	6 months (*n* = 37)	*p*-Value ^a^	Baseline (*n* = 50)	6 months (*n* = 39)	*p*-Value ^a^	*Baseline* *p*-Value ^b^	∆ *p*-Value ^c^
AST (IU/L)	25.5 (11.0)	21.6 (6.1)	<0.001	23.9 (8.3)	21.9 (8.5)	0.302	0.487	0.116
ALT (IU/L)	33.1 (16.8)	22.9 (8.5)	<0.001	33.3 (17.9)	21.7 (9.2)	<0.001	0.759	0.474
GGT (IU/L)	40.9 (29.0)	28.3 (23.0)	<0.001	33.6 (23.9)	26.4 (42.6)	<0.001	0.174	0.692
Hepatic volume (mL)	1797 (433)	1633 (316)	<0.001	1758 (406)	1563 (330)	<0.001	0.721	0.636
Liver fat (%)	7.4 (5.3)	3.8 (3.3)	<0.001	7.0 (5.4)	2.8 (3.1)	<0.001	0.468	0.706
Liver stiffness (m/s)	1.9 (0.8)	2.0 (0.7)	0.177	1.8 (0.8)	1.7 (0.6)	0.203	0.588	0.062
FLI	80.4 (15.6)	54.4 (23.7)	<0.001	76.9 (21.2)	47.9 (24.1)	<0.001	0.654	0.123

Data are presented as the mean (SD). Nonalcoholic fatty liver disease (NAFLD). American Health Association (AHA). Fatty Liver in Obesity (FLiO). Asparate aminotransferase (AST). Alanine aminotransferase (ALT). Gamma-glutamyl transferase (GGT). Fatty Liver Index (FLI). ^a^ Comparison within dietary groups (baseline and after 6 months). ^b^ Baseline differences between the AHA and FLiO groups. ^c^ Comparison of the changes (baseline and 6 months) between the AHA and FLiO groups.

**Table 3:** Dietary intake and physical activity at baseline and after 6 months of dietary intervention in subjects with NAFLD according to the group of intervention.

	AHA			FLiO					
	Baseline (*n* = 48)	6 months (*n* = 37)	*p*-Value ^a^	Baseline (*n* = 50)	6 months (*n* = 39)	*p*-Value ^a^	Baseline *p*-Value ^b^	∆ *p*-Value ^c^	6 months *p*-Value ^d^
Total energy (kcal/day)	2730 (867)	2170 (474)	0.002	2521 (1000)	1816 (569)	<0.001	0.148	0.536	0.012
Meal frequency	4.5 (0.9)	4.8 (0.8)	0.045	4.8 (0.9)	5.8 (1.0)	<0.001	0.199	0.022	0.001
Carbohydrates (% TEV)	43.4 (7)	45.0 (7)	0.336	42.8 (7)	39.0 (7)	0.015	0.685	0.048	<0.001
Proteins (% TEV)	16.8 (3)	18.5 (3)	0.047	17.6 (4)	22.1 (4)	<0.001	0.285	0.018	<0.001
Lipids (% TEV)	37.0 (7)	34.5 (6)	0.111	36.9 (8)	36.5 (9)	0.973	0.927	0.444	0.292
MUFA (% TEV)	17.8 (5)	17.5 (4)	0.682	17.4 (4)	16.1 (6)	0.353	0.785	0.623	0.088
PUFA (% TEV)	5.5 (2)	5.2 (2)	0.704	5.4 (2)	9.0 (5)	0.001	0.727	0.001	<0.001
SFA (% TEV)	10.6 (2)	9.4 (2)	0.032	10.6 (3)	8.8 (3)	0.024	0.838	0.572	0.102
Fiber (g/1000 kcal)	9.8 (3)	14.4 (4)	<0.001	9.7 (4)	14.9 (4)	<0.001	0.586	0.380	0.577
Glycemic load	165 (78)	117 (41)	0.003	147 (78)	83.8 (34)	<0.001	0.181	0.667	<0.001
TAC (mmol/1000 kcal)	4.9 (2)	4.6 (2)	0.426	4.2 (2)	5.7 (3)	0.022	0.332	0.044	0.264
MedDiet adherence score	6.2 (2)	10.8 (3)	<0.001	5.8 (2)	12.6 (3)	<0.001	0.370	0.002	0.002
PA (METs—min/week)	2685 (2112)	4463 (3031)	<0.001	3120 (2136)	4158 (2532)	0.039	0.258	0.326	0.809
Ratio Energy intake/Energy expenditure in PA	7.1 (6)	3.0 (2)	0.001	5.8 (7)	2.7 (2)	0.033	0.088	0.298	0.502

Data are presented as the mean (SD). Nonalcoholic fatty liver disease (NAFLD). American Health Association (AHA). Fatty Liver in Obesity (FLiO). Total energy value (TEV). Monounsaturated fatty acid (MUFA). Polyunsaturated fatty acid (PUFA). Saturated (SFA). Total antioxidant capacity (TAC). Mediterranean diet (MedDiet). Physical Activity (PA). Metabolic equivalent of the task (METs). ^a^ Comparison within dietary groups (baseline and after 6 months). ^b^ Differences between the AHA and FLiO groups at baseline. ^c^ Comparison of the changes (baseline and 6 months) between the AHA and FLiO groups. ^d^ Differences between the AHA and FLiO groups at 6 months of intervention.

**Table 4:** Regression analyses of the hepatic status parameters after 6 months of dietary intervention as dependent variables and changes in anthropometric, biochemical, and dietary factors as independent variables.

	Weight Loss (%)		Δ Visceral Adipose Tissue (kg)		Δ Total Fat Mass (%)		Δ Adiponectin (µg/mL)		Δ C-Reactive Protein (mg/dL)		Δ MedDiet Adherence		Δ Proteins (%)		Δ Lipids (%)		Δ Meal Frequency		Δ TAC (mmol/1000 kcal)		Δ Fiber (g/1000 kcal)		Δ Glycemic Load	
	*β*	*p*	*β*	*p*	*β*	*p*	*β*	*p*	*β*	*p*	*β*	*p*	*β*	*p*	*β*	*p*	*β*	*p*	*β*	*p*	*β*	*p*	*Β*	*p*
AST (IU/L)	−0.191	0.263	1.672	0.059	0.086	0.742	0.151	0.565	3.389	0.224	−0.307	0.320	−0.096	0.682	0.124	0.272	−0.325	0.806	−0.642	0.194	0.001	0.995	0.008	0.531
ALT (IU/L)	−0.369	0.046	1.580	0.089	0.311	0.260	−0.010	0.972	6.991	0.021	−0.626	0.062	−0.093	0.719	0.191	0.123	−0.062	0.964	−0.287	0.602	−0.246	0.372	−0.001	0.906
GGT (IU/L)	−0.691	0.113	0.785	0.745	1.129	0.095	0.117	0.862	13.146	0.089	−0.799	0.312	−0.242	0.699	−0.314	0.292	0.085	0.981	−0.749	0.572	−1.112	0.103	0.029	0.391
Hepatic Volume (mL)	−5.489	0.450	15.68	0.664	1.503	0.899	−3.840	0.731	162.95	0.096	−3.444	0.770	2.780	0.761	3.543	0.437	−41.099	0.320	0.388	0.984	0.888	0.931	−0.597	0.274
Liver fat (%)	−0.252	<0.001	0.123	0.753	0.338	0.004	−0.166	0.080	1.377	0.174	−0.396	0.001	−0.111	0.268	0.098	0.046	−0.227	0.549	−0.238	0.282	−0.155	0.167	0.005	0.369
FLI	−2.340	<0.001	3.402	0.078	3.590	<0.001	−0.1.45	0.069	11.966	0.199	−0.962	0.313	−0.308	0.668	0.319	0.353	−5.156	0.172	−0.391	0.795	−1.872	0.015	0.018	0.645
Liver Stiffness (m/s)	−0.043	0.004	0.168	0.044	0.070	0.003	−0.010	0.679	0.140	0.589	−0.053	0.061	0.016	0.451	−0.009	0.375	−0.147	0.214	−0.006	0.887	−0.034	0.143	−0.0002	0.980

Models were adjusted by diet (group of intervention), age, sex, 6 months physical activity, and 6 months energy intake. Mediterranean diet (MedDiet). Total antioxidant capacity (TAC). Asparate aminotransferase (AST). Alanine aminotransferase (ALT). Gamma-glutamyl transferase (GGT). Fatty Liver Index (FLI).

**Table 5:** Regression analyses of liver fat percentage at 6 months as the dependent variable and MedDiet adherence, total antioxidant capacity, and weight loss percentages after the dietary intervention as independent variables.

Liver Fat (%) after 6 Months of Treatment		*β*	*p*	Adjusted *R*^2^	*p*-Model
Unadjusted model	6 months MedDiet adherence	−0.465	<0.001	0.186	<0.001
					
Model 1	6 months MedDiet adherence	−0.585	<0.001	0.319	<0.001
					
Unadjusted model	6 months TAC (mmol/1000 kcal)	−0.351	0.028	0.061	0.027
					
Model 2	6 months TAC (mmol/1000 kcal)	−0.306	0.102	0.114	0.047
					
Unadjusted model	Weight loss (%)	−0.257	<0.001	0.188	<0.001
					
Model 3	Weight loss (%)	−0.252	<0.001	0.259	<0.001
					
Model 4	Weight loss (%)			0.366	<0.001
	5%–10%	−3.410	0.001		
	>10%	−5.033	<0.001		
					
Model 5	6 months MedDiet adherence	−0.437	0.007	0.344	<0.001
	Weight loss (%)	−0.133	0.087		
					
Model 6	6 months MedDiet adherence	−0.325	0.031	0.409	<0.001
	Weight loss (%)				
	5%–10%	−2.490	0.021		
	>10%	−3.638	0.002		
					
Model 7	6 months TAC (mmol/1000 kcal)	−0.351	0.037	0.305	<0.001
	Weight loss (%)	−0.262	<0.001		
					
Model 8	6 months TAC (mmol/1000 kcal)	−0.297	0.056	0.398	<0.001
	Weight loss (%)				
	5%–10%	−3.410	0.001		
	>10%	−5.009	<0.001		

All models were adjusted by dietary group (group of intervention), age, sex, 6 months physical activity and 6 months energy intake. Mediterranean diet (MedDiet). Total antioxidant capacity (TAC).

## Supplementary Materials

The following are available online at https://www.mdpi.com/2072-6643/11/10/2543/s1, Table S1: Regression analyses of liver fat percentage at 6 months as dependent variable and selected dietary components after the dietary intervention as independent variables.
