# Supplementary material for: The Metabolic and Hepatic Impact of Two Personalized Dietary Strategies in Subjects with Obesity and Nonalcoholic Fatty Liver Disease: The Fatty Liver in Obesity (FLiO) Randomized Controlled Trial
Source: Nutrients. 2019 Oct 22;11(10):2543. doi: 10.3390/nu11102543 (PMC6836315; doi:10.3390/nu11102543)
Supplement: Supplementary file 1 [file nutrients-11-02543-s001.pdf]

Table S1. Regression analyses of liver fat percentage at 6 months as dependent variable and selected dietary components after the dietary intervention as independent variables.

**6-month Liver Fat (%)**		**β**	***p***	Adjusted R^2	*p*-model
**Unadjusted model**	6-month TAC (mmol/1000kcal)	*-0.351*	0.028	0.061	0.027
**Model 1**	6-month TAC (mmol/1000kcal)	-0.351	0.037	0.305	<0.001
	Weight loss (%)	-0.262	<0.001		
**Model 2**	6-month TAC (mmol/1000kcal)	-0.297	0.056	0.398	<0.001
	Weight loss (%)				
	5-10%	-3.410	0.001		
	>10%	-5.009	<0.001		
**Unadjusted model**	6-month fiber (g/1000kcal)	*-0.189*	0.084	0.031	0.084
**Model 1**	6-month fiber (g/1000kcal)	*-0.075*	0.170	0.272	<0.001
	Weight loss (%)	*-0.237*	0.001		
**Model 2**	6-month fiber (g/1000kcal)	*-0.037*	0.469	0.360	<0.001
	Weight loss (%)				
	5-10%	*-3.281*	0.002		
	>10%	*-4.826*	<0.001		
**Unadjusted model**	6-month Proteins (% TEV)	*-0.145*	0.175	0.013	0.175
**Model 1**	6-month Proteins (% TEV)	*0.029*	0.827	0.246	0.002
	Weight loss (%)				
**Model 2**	6-month Proteins (% TEV)	*-0.004*	0.970	0.354	<0.001
	Weight loss (%)				
	5-10%	*-3.411*	0.001		
	>10%	*-5.026*	<0.001		
**Unadjusted model**	6-month Lipids (% TEV)	*0.090*	*0.097*	0.028	0.096
**Model 1**	6-month Lipids (% TEV)	*0.090*	0.127	0.278	<0.001
	Weight loss (%)	*-0.231*	0.001		
**Model 2**	6-month Lipids (% TEV)	*0.046*	0.417	0.362	<0.001
	Weight loss (%)				
	5-10%	*-3.202*	0.003		
	>10%	*-4.758*	<0.001		
**Unadjusted model**	6-month MUFA (% TEV)	*0.146*	0.069	0.037	0.069
**Model 1**	6-month MUFA (% TEV)	*0.097*	0.272	0.262	0.001
	Weight loss (%)	*-0.228*	0.002		
**Model 2**	6-month MUFA (% TEV)	*0.052*	0.534	0.359	<0.001
	Weight loss (%)				
	5-10%	*-3.300*	*0.002*		
	>10%	*-4.801*	<0.001		
**Unadjusted model**	6-month PUFA (% TEV)	*-0.101*	0.300	0.001	0.300
**Model 1**	6-month PUFA (% TEV)	*-0.060*	0.590	0.249	0.001
	Weight loss (%)	*-0.250*	0.001		
**Model 2**	6-month PUFA (% TEV)	*-0.144*	0.172	0.377	<0.001
	Weight loss (%)				
	5-10%	*-3.740*	0.001		
	>10%	*-5.173*	<0.001		
**Unadjusted model**	6-month SFA (% TEV)	*0.305*	0.032	0.056	0.032
**Model 1**	6-month SFA (% TEV)	*0.219*	0.100	0.283	<0.001
	Weight loss (%)	*-0.249*	<0.001		

**Model 2**	6-month SFA (% TEV)	*0.167*	0.179	0.376	<0.001
	Weight loss (%)				
	5-10%	*-3.340*	0.001		
	>10%	*-4.887*	<0.001		

Model 1 and model 2 were adjusted by dietary group (group of intervention), age, sex, 6-month physical activity and 6-month energy intake. Total energy value (TEV). Monounsaturated fatty acid (MUFA). Polyunsaturated fatty acid (PUFA). Saturated (SFA). Total antioxidant capacity (TAC).
